# Supplementary material for: Fuzzy Overclustering: Semi-Supervised Classification of Fuzzy Labels with Overclustering and Inverse Cross-Entropy
Source: Sensors (Basel). 2021 Oct 7;21(19):6661. doi: 10.3390/s21196661 (PMC8512301; doi:10.3390/s21196661)
Supplement: Supplementary file 1 [file sensors-21-06661-s001.zip › sensors-1373615-supplementary/supple.pdf]

sensors

MDPI

Article

Fuzzy Overclustering: Semi-supervised classification of fuzzy labels with overclustering and inverse cross-entropy - Supplementary Material

Lars Schmarje[1], Johannes Brünger [1], Monty Santarossa [1], Simon-Martin Schröder [1], Rainer Kiko[2] and Reinhard Koch [1]

[1] Multimedia Information Processing Group, Kiel University, Germany; {las, jbr, msa, sms, rk}@informatik.uni-kiel.de
[2] Laboratoire d'Océanographie de Villefranche, Sorbonne Université, France; rainer.kiko@obs-vlfr.fr
* Correspondence: las@informatik.uni-kiel.de

check for updates

Citation: Schmarje, L.; Brünger, J.; Santarossa, M., Schröder, S.-M.; Kiko, R.; Koch, R. Fuzzy Overclustering: Semi-supervised classification of fuzzy labels with overclustering and inverse cross-entropy - Supplementary Material. *Sensors* **2021**, *21*, 6661. https://doi.org/10.3390/s21196661

Received: 24 August 2021
Accepted: 2 October 2021
Published: 7 October 2021

Publisher's Note: MDPI stays neutral with regard to jurisdictional claims in published maps and institutional affiliations.

Copyright: © 2020 by the authors. Licensee MDPI, Basel, Switzerland. This article is an open access article distributed under the terms and conditions of the Creative Commons Attribution (CC BY) license (https://creativecommons.org/licenses/by/4.0/).

1. Fuzzy Overclustering

1.1. Proof for Cross-Entropy Inverse

We claimed in our paper that we would minimize the entropy $H(\Phi(x_1))$ if we minimize $CE^{-1}(\Phi(x_1), \Phi(x_3))$. For two discrete probability distributions p, q over all classes C, this claim is trivial due to the fact that $CE(p,q) = H(p) + K(p,q)$ with K Kullback-Leibler divergence. However, $\Phi(x_3)$ might not be a probability distribution. We show that the equation still holds for p a probability distribution and $q(c) \in [0,1]$ for all $c \in C$.

$$\begin{aligned} CE(p,q) &= -\sum_{c\in C} p(c) \cdot log(q(c)) \\ &= -\sum_{c\in C} p(c) \cdot log(\frac{p(c) \cdot q(c)}{p(c)}) \\ &= -\sum_{c\in C} p(c) \cdot [log(p(c)) + log(\frac{q(c)}{p(c)})] \\ &= -\sum_{c\in C} p(c) \cdot log(p(c)) + -\sum_{c\in C} p(c) \cdot log(\frac{q(c)}{p(c)}) \\ &= H(p) + K(p,q) \end{aligned} \tag{1}$$

1.2. Restricted Unsupervised Data

As stated in the paper, we restrict the unlabeled data by the fixed ratio r. We restrict the unlabeled every batch and therefore the unlabeled data per epoch. The ratio r is an upper bound for the unlabeled data and less unlabeled data can be used in each batch if it is not available. In Table 1, we give several examples of how much unsupervised data is used, especially in borderline cases.

2. Implementation Details

In this section, we report additional hyperparameters.

We parallelize the processing of the input instead of the sequential approach of IIC. This leads to a higher memory usage which we counter by decreasing the repetition of images per batch to 3 when using the MI part of the loss. Only the overclustering head uses the additional unlabeled data. We train the framework either on a Titan XP or an RTX 2080 Ti while the majority is trained on the RTX. In our environment, both GPUs perform similarly regarding speed and have about 12GB of VRAM. If we need more VRAM we used multiple GPUs in parallel. The batchsize varied depending on the number of

	Total		Batch	
r	Labeled	Unlabeled	Labeled	Unlabeled
0	100	0	100	0
0	10	90	100	0
0	90	10	100	0
0	50	50	100	0
0.5	100	0	100	0
0.5	10	90	50	50
0.5	90	10	90	10
0.5	50	50	50	50
1.0	100	0	100	0
1.0	10	90	10	90
1.0	90	10	90	10
1.0	50	50	50	50

Table 1: Examples of restricted unsupervised data – The first column describes the ratio r. All other columns are the percentage of used labeled or unlabeled data relative to the total number of images or the batch size.

repetitions of the images per batch. The MI part of the loss is calculated over the complete batch and therefore the batchsize should be at least about the number of output clusters times repetition. In the unsupervised case, no third input and repetition of 3 is used. We need 24GB VRAM for a batchsize of 195. In the supervised case, we need 36GB VRAM for a batchsize of 390. For some ablations, we do not use the MI part of the loss and repetition of 1. We need 12GB of VRAM for a batchsize of 130. During the training, we lower the learning rate after 100 epochs by factor 10 if no improvement is visible based on the proposed metrics in the paper. We stop the training if no improvement is visible for a third of all epochs.

For the augmentations, we use imgaug[1]. The source code of the used augmentations is given in Listing 1. The `target_size` is a tuple with the target cropping width and height. We use `train_crop` as augmentation g_1 in the pretext task and `train_aug_affine_cutout` in the fine-tuning. We use `train_augment_mi` as augmentation g_2 and `val_crop` as g_3. For evaluation, we use `val_crop` for all augmentations.

Listing 1: Used imgaug augmentations

```
def train_crop(target_size):
  return iaa.Sequential([
    iaa.CropToFixedSize(
      width=target_size[0],
      height=target_size[1],
      position="uniform"),
  ])

def train_augment_mi(target_size):
  return iaa.Sequential([
    iaa.CropToFixedSize(
                width=target_size[0],
        height=target_size[1],
        position="uniform"),
    iaa.Fliplr(0.5),
    iaa.AddToHue((-30,30)),
```

[1] https://github.com/aleju/imgaug

```
        iaa.AddToSaturation((-100,100)),
        iaa.GammaContrast(gamma=(0.6,1.4))
    ])

def train_aug_affine_cutout(target_size):
    return iaa.Sequential([
        iaa.CropToFixedSize(
            width=target_size[0],
            height=target_size[1],
            position="uniform"),
        iaa.Fliplr(0.5),
        iaa.MultiplyHue(mul=(1-0.125,1.125)),
        iaa.MultiplySaturation(mul=(0.6,1.4)),
        iaa.Multiply(mul=(0.6,1.4)),
        iaa.GammaContrast(gamma=(0.6,1.4)),
        iaa.Cutout(
            nb_iterations=1,
            fill_mode="constant",
            cval=0,
            size=(0.2,0.7)),
        iaa.Affine(
            rotate=(-20, 20),
            shear=(-10, 10),
            order=1,
            cval=0,
            mode='constant'
            )
        ])


def val_crop(target_size):
    return iaa.Sequential([
        iaa.CropToFixedSize(
            width=target_size[0],
            height=target_size[1],
            position="center"),
    ])
```

3. Plankton dataset

3.1. Dataset generation

In this subsection, we describe the creation of the plankton dataset in more detail. As mentioned in the paper, PlanktonID [2] was used to create multiple annotations with the help of citizen scientists. We use the data generated by version two of PlanktonID. This version is only available to users which have done at least 1000 annotations in version one. Therefore, we can assume that the annotators know the differences between the classes and are dedicated to creating consistent results. The second version of PlanktonID is a game where example images can be sorted into three proposed classes. If none of the proposed classes seem to fit, the user has the option to select any other of the 28 classes[3] of PlanktonID (including a 'no fitting category' class). The initial proposals are generated by a neural network, hence, a confirmation bias might be introduced.

Some classes of the PlanktonID dataset have very few examples in comparison to others (e.g. three single images). For this semi-supervised algorithm in contrast to few-shot learning we picked the largest classes and merged some smaller classes (e.g. different classes of detritus). All other images are assigned to the class 'no fitting category'. The

[2] https://planktonid.geomar.de/en
[3] https://planktonid.geomar.de/en/classes

usage of the 'no fitting category' class has two main reasons. With this class, we can check how our system performs with a skewed class distribution and a mixture of distinct subclasses. Moreover, a rescaling of the `fuzzy` labels is needed if some classes are excluded from the original assignments. This rescaling leads to a loss of interpretability of the `fuzzy` labels by humans. A complete overview of the mapping is given in Table 2. As mentioned in the paper, we call images `certain` if the annotations are consistent among all users and `fuzzy` if at least one annotation is different. We use only `certain` images for training and validation. We aim for 400 training and 200 validation images per class. If not enough `certain` images are available, we use random duplicates. The `fuzzy` labeled images and not used `certain` images are used as unlabeled data. The exact number of used images per class is shown in Table 3. An illustration of the distribution of the data is given in Figure 1.

Label PlanktonID	Assigned Label
Bubbles	Bubbles
Detritus fluffy - dark	Detritus
Detritus compact	Detritus
Detritus fluffy - light	Detritus
Detritus feces like	Detritus
Trichodesmium Tuft	Trichodesmium Tuft
Trichodesmium Puff	Trichodesmium Puff
Rhizaria Phaeodaria Leg	Rhizaria Phaeodaria
Rhizaria Phaeodaria Sphere Eye	Rhizaria Phaeodaria
Rhizaria Phaeodaria sphere thorn	Rhizaria Phaeodaria
Collodaria black	Collodaria black
Collodaria globule	Collodaria globule
Crust copepod	Crust copepod
Shrimp like	Shrimp like
All other 14 categories	No fitting category

Table 2: Mapping of labels for the plankton data

Label	`certain`	`fuzzy`	total
No fitting category	3073	656	3729
Bubbles	298	213	511
Detritus	264	788	1052
Trichodesmium Puff	207	367	574
Trichodesmium Tuft	916	687	1603
Rhizaria Phaeodaria	995	124	1119
Collodaria black	671	174	845
Collodaria globule	565	136	701
Crust copepod	1194	208	1402
Shrimp like	522	222	744
sum	8705	3575	12280

Table 3: Amount of data per class in the plankton data

3.2. Consistency evaluation

In Table 4 and Table 5 we give the number of consistent images per cluster which were used to calculate the consistency scores in table 3 in our paper. The tables have different a number of rows because FixMatch [1] does not use overclustering. The total number of images without the no-fit class can not be calculated from these tables. It is given in Table 3 including the train and validation images. The mean and standard deviation of the consistency per cluster are calculated based on the scores provided in the tables.

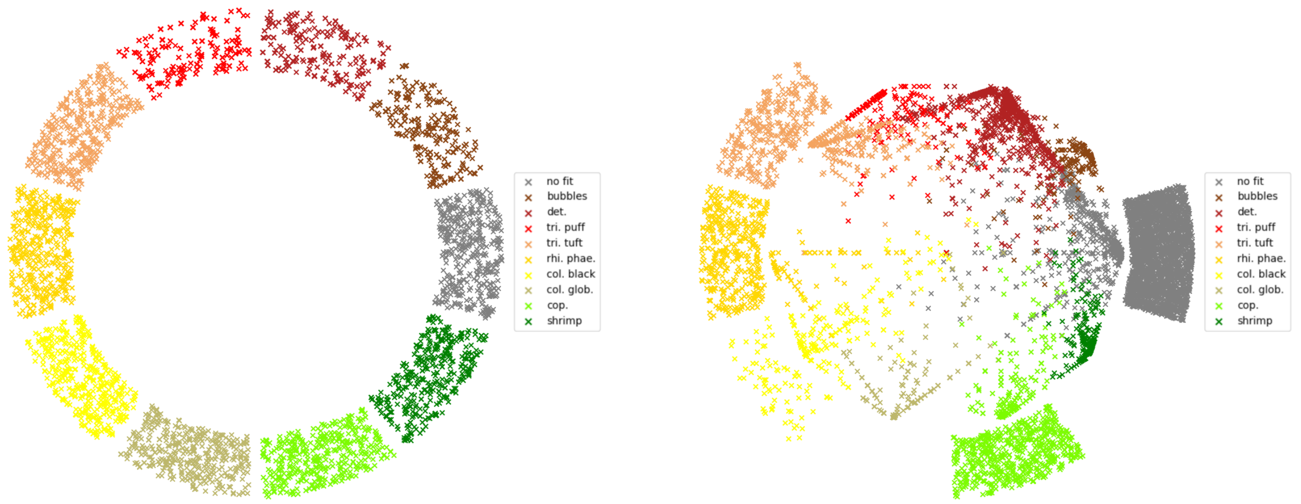

(a) training / validation **(b)** unlabeled

Figure 1. Illustration of class distribution for the plankton dataset – Every cross represents an image while the color indicates the class label. All `certain` images are arranged at random positions within a ring segment for each class. These ring segments are clearly visible on the left-hand side for the training or validation data since only `certain` images are used. The `fuzzy` labeled images of the unlabeled data are shown as interpolation of the different classes depending on their `fuzzy` label. For example, an image with label `det. 0.5 tri.tuft 0.5` would be directly in the center. This representation leads to a loss of information and therefore a reconstruction of the original data points is not possible.

# images	# consistent images	cluster consistency	note
3073	2817	91.67	class no-fit
198	180	90.91	
184	183	99.46	
393	392	99.75	
1079	860	79.70	
567	558	98.41	
213	202	94.83	
443	87	19.64	
857	734	85.65	
417	116	27.28	
7424	6129	82.56	total
4295	3312	77.11	total without class no-fit

Table 4: Consistency per predicted cluster for FixMatch – Each row describes a predicted cluster with the number of images, the number of consistent images in that cluster, the consistency for the cluster and additional notes. At the end, a sum over the complete data and the data without the no-fit class is provided.

4. SYN-CE

We give an example for the calculation of the `fuzzy` label for a bubble. A bubble with a hue of 60 and an axis ratio of 1.3 has an interpolated color of $p_c = (0.5, 0.5, 0)$ for the classes red (r), green (g) and blue (b). The interpolated geometry is $p_g = (0.7, 0.3)$ for the classes circle (c) and ellipse (e). The combined `fuzzy` label for the classes rc, gc, bc, re, ge and be is $l = (0.35, 0.35, 0, 0.15, 0.15, 0)$.

In Figure 2 we present 12 examples of the SYN-CE dataset.

# images	# consistent images	cluster consistency	note
166	158	95.18	
194	181	93.29	
1	1	100.00	
89	82	92.13	
6	3	50.00	class no-fit
50	30	60.00	
845	780	92.31	
108	90	83.33	
2645	2556	96.64	class no-fit
3	3	100.00	
7	6	85.71	
268	209	77.99	
260	149	57.31	class no-fit
487	482	98.97	
90	87	96.67	
292	287	98.29	
83	33	39.76	class no-fit
342	160	46.78	
523	460	87.95	
158	128	81.01	
113	94	83.19	
34	20	58.82	class no-fit
567	469	82.72	
93	50	53.76	class no-fit
7424	6518	87.80	total
4295	3707	86.31	total without class no-fit

Table 5: Consistency per predicted cluster for FOC – Each row describes a predicted cluster with the number of images, the number of consistent images in that cluster, the consistency for the cluster and additional notes. At the end, a sum over the complete data and the data without the no-fit class is provided.

5. Additional Results

5.1. Results on STL-10

We present further analysis of the STL-10 dataset in this section. Following previous literature [2,3], we evaluate the unsupervised clustering on the complete labeled dataset if we use no fine-tuning. In this case, we report the results for the head with the lowest loss. The parts of FOC which use label information are not used in unsupervised clustering e.g. supervised augmentations. In the case of unsupervised clustering, IIC and FOC are quite similar apart from the used heads and the restriction on unsupervised data.

On the warm-up on STL-10, we investigate the impact of r on the runtime and the benefit of supervised augmentations in Table 6. The direct comparison between IIC and FOC ($r = 1.0$) shows that we outmatch IIC by 1.34%. Because the reported results of IIC used a higher number of clusters than ours, we conducted our own experiment with an equal number of clusters. Our restricted FOC ($r = 0.5$) performs almost equally to this better comparable version of IIC. Due to the restriction of unlabeled data, our method has a lower runtime by the factor of about 9. As a comparison, we added FOC with $r = 1.0$ and supervised augmentations as an upper bound estimation. We can identify a clear gap of 8 to 20% in the performance with this low form of supervision.

A comparison between IIC [2] and our method regarding accuracy and runtime is given in Table 7. Our method FOC ($r = 1.0$) can achieve even slightly better results due to

Table 6: Warm-up on STL-10 – r indicates the restriction of unlabeled data. The runtime is the training time in hours on the same hardware. The best results are marked bold. A horizontal line indicates different comparisons. Legend: * Original authors code used. † Uses supervised augmentations and can be seen as upper bound estimation.

		Accuracy		
Method	r	Overcluster	Normal	Runtime
IIC [2]	-	63.1		–
IIC*	-	59.31		114 h
FOC	0	57.28	48.10	**10 h**
FOC	0.5	59.24	**52.96**	13 h
FOC	1	**64.44**	50.92	89 h
FOC†	1	72.06	69.83	81 h

	Accuracy		
Method	Overcluster	Normal	Runtime
IIC [2]		59.6	ca. 216 h[4]
FOC ($r = 0.5$)	(62.45)	55.99	40 h
FOC ($r = 1.0$)	(65.76)	60.45	93 h

Table 7: Comparison FOC and IIC on unsupervised image classification on STL-10

advanced hyperparameters such as augmentations. The real improvement with FOC is in the runtime. It is difficult to compare runtimes between different setups. We even use two GPUs instead of one to achieve 24GB of VRAM like [2]. However, the magnitude of change is clearly visible. We parallelize the feed forward pass through our network and can cut the runtime by more than a half. In combination with restricted unlabeled data ($r = 0.5$), we can achieve slightly worse results with a speed-up factor of 5 compared to the original IIC.

We further investigate the benefits of each part of the loss in our framework in Table 8 with a ResNet50v2 backbone and ImageNet weights. The first row represent the supervised baseline that we trained only with cross-entropy and without an unsupervised pretext task. The accuracy on the normal head improves slightly for each part of the loss which is added and if unlabeled data is used. Overall the accuracy increases from around 83% to 85%. However, the changes on the overclustering head make a difference from around 30%. The biggest changes are the usage of an unsupervised pretext task and CE^{-1}or MI. One additional benefit of $CE^{-1}$to MI which is not visible in the table is that CE^{-1}independent of the batch size. Therefore, it can still be used with less memory available.

5.2. Results on plankton dataset

In Figure 3, we illustrate the predictions of our framework of the unlabeled data in the style of Figure 1. If we compare clusters 03 and 04 of the normal head with clusters 10, 41 and 47 of the overclustering head we see the benefit of overclustering. The clusters overlap for the normal head but for the overclustering head the cluster 47 holds a mixture of both classes.

6. Qualitive Results

6.1. Certain Training Examples

This section shows 12 random samples of each class in the training data. The corresponding figures are : Figure 4, Figure 5, Figure 6, Figure 7, Figure 8, Figure 9, Figure 10, Figure 11, Figure 12,Figure 13.

Method	Pre	MI	CE^{-1}	Use Unlabeled Data	Accuracy	
					Overcluster	Normal
FOC					58.55	84.11
FOC	X				74.95	83.66
FOC	X		X		80.85	83.80
FOC	X	X			82.03	84.48
FOC	X	X		X	82.74	84.54
FOC	X	X	X		82.44	85.25
FOC	X	X	X	X	83.70	85.25

Table 8: Ablation study of semi-supervised results for STL-10 with a ResNet50v2 Backbone and ImageNet initialization

6.2. Fuzzy Unlabeled Examples

This section shows 12 random samples of unlabeled data for all predicted clusters with at least 12 samples. The corresponding figures are : Figure 14, Figure 15, Figure 16, Figure 17, Figure 18, Figure 19, Figure 20, Figure 21, Figure 22, Figure 23, Figure 24, Figure 25, Figure 26, Figure 27, Figure 28, Figure 29, Figure 30, Figure 31, Figure 32, Figure 33.

[4] Official Repo https://github.com/xu-ji/IIC/issues/4

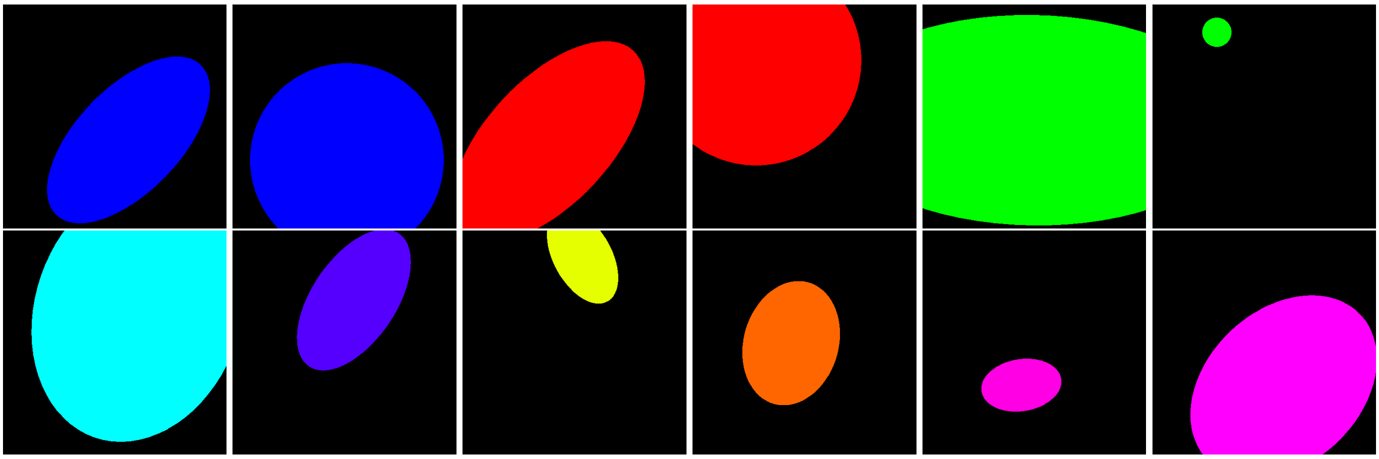

Figure 2. 12 Examples of the SYN-CE dataset – The upper half are `certain` examples and the lower have are `fuzzy` examples.

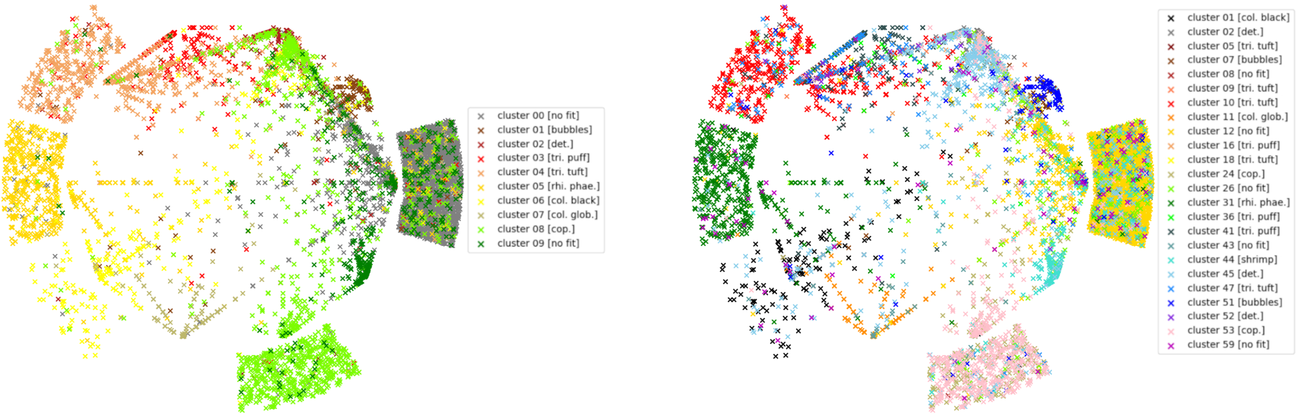

(a) normal head **(b)** overcluster head

Figure 3. Prediction of clusters – Every cross represents an image while the color indicates the cluster number. The most likely class label for each cluster is given in brackets based on the `fuzzy` labels. Due to the usage of cross-entropy for the normal head, the clusters correspond to the classes. This is not the case for the overclustering head as you can see based on the different color codes.

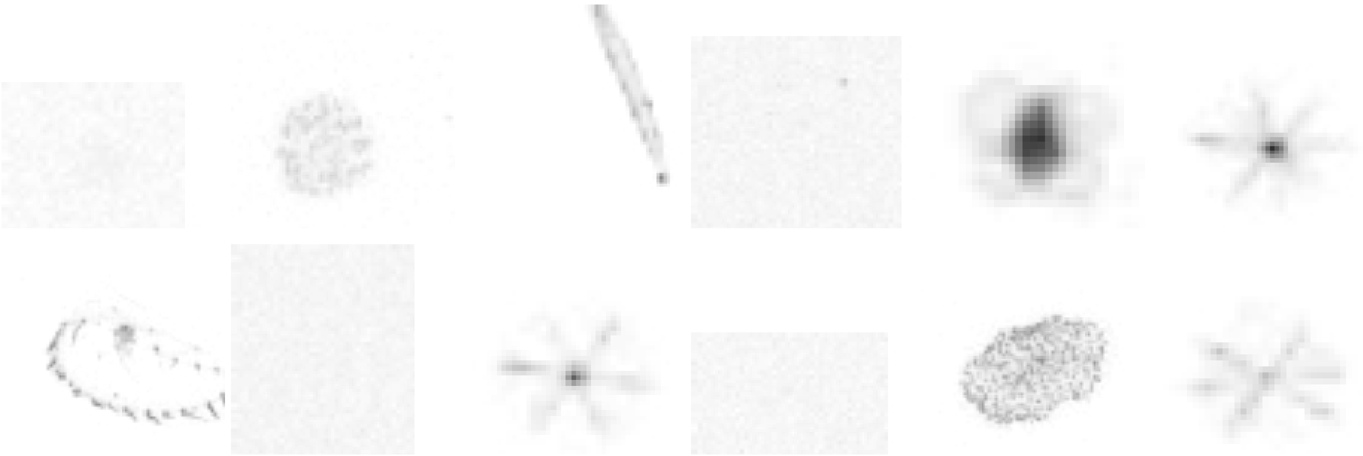

Figure 4. Label: no fitting category

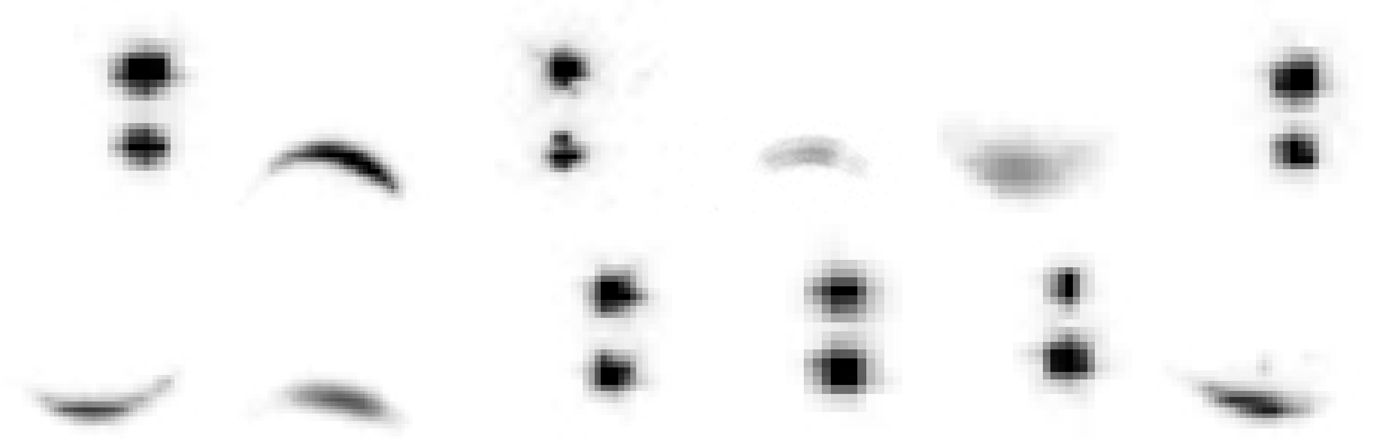

Figure 5. Label: bubbles

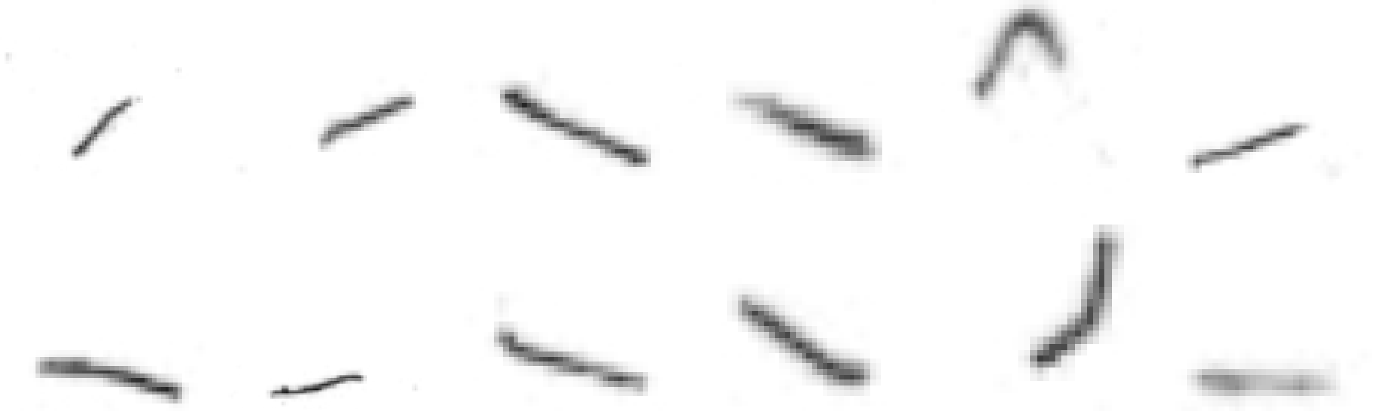

Figure 6. Label: detritus

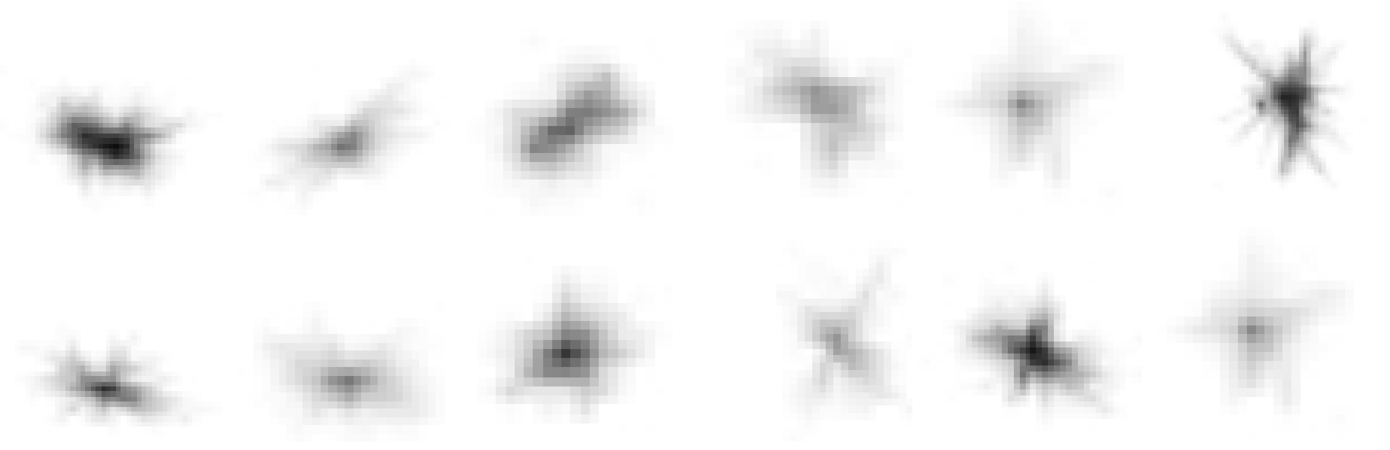

Figure 7. Label: trichodesmium puff

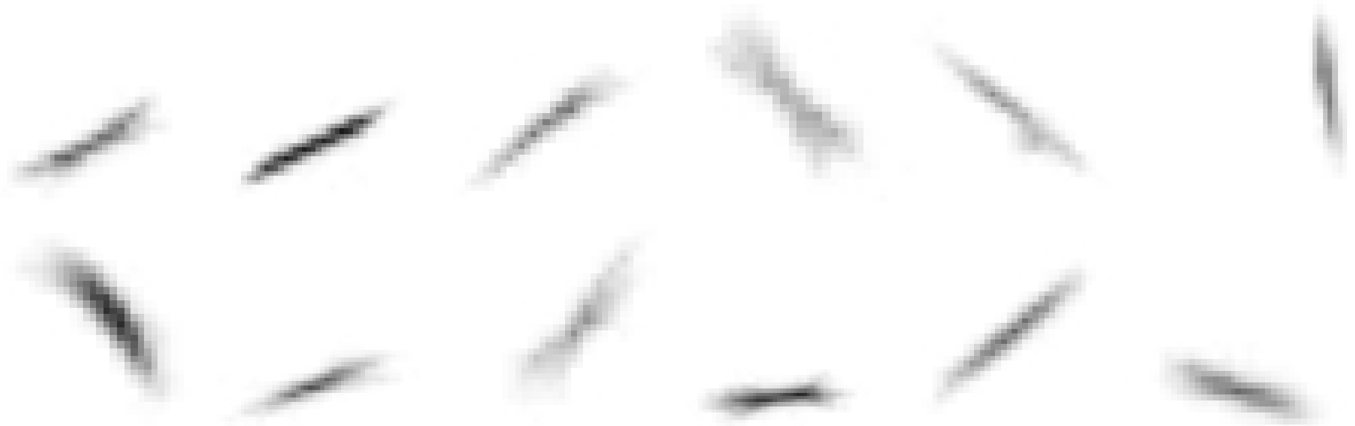

Figure 8. Label: trichodesmium tuft

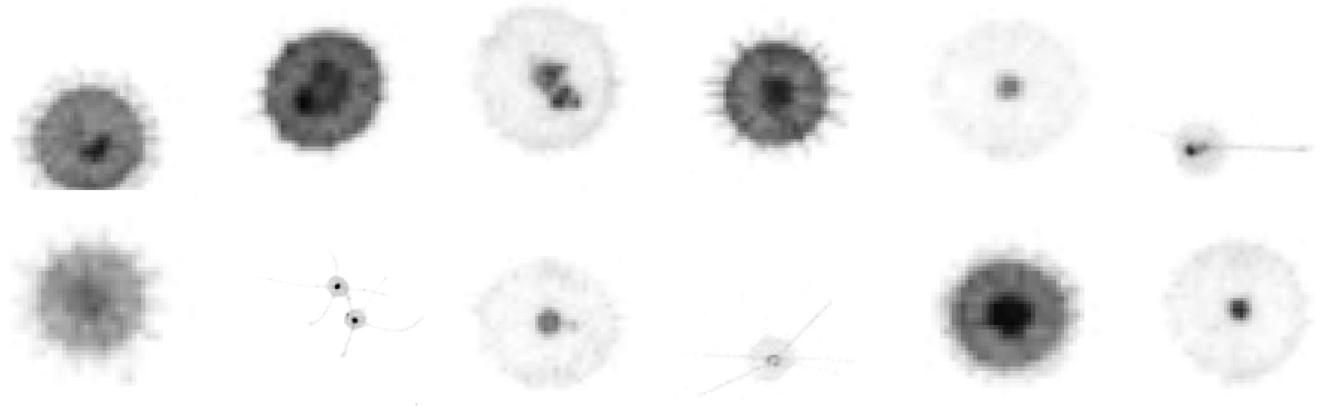

Figure 9. Label: Rhizaria Phaeodaria

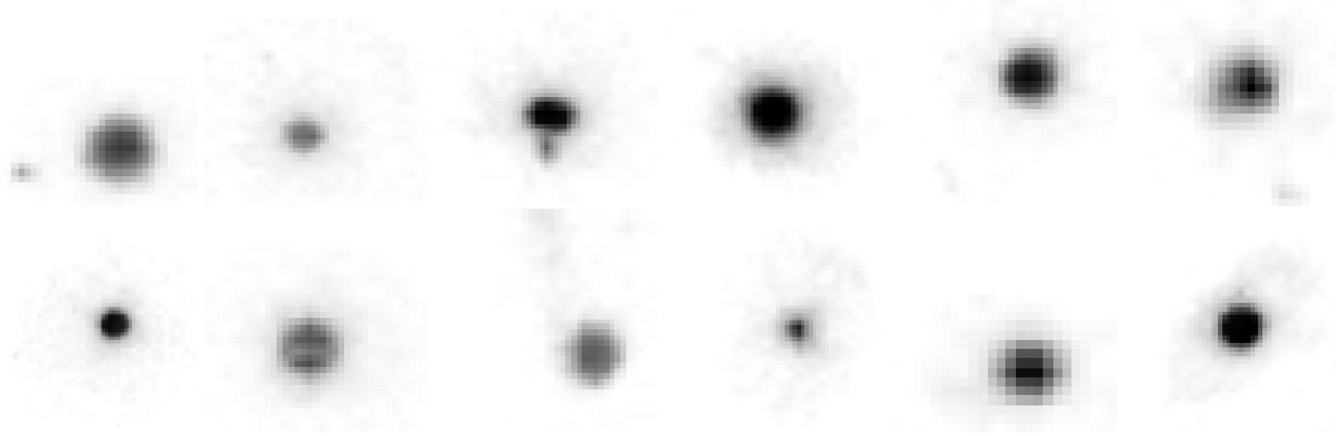

Figure 10. Label: Collodaria black

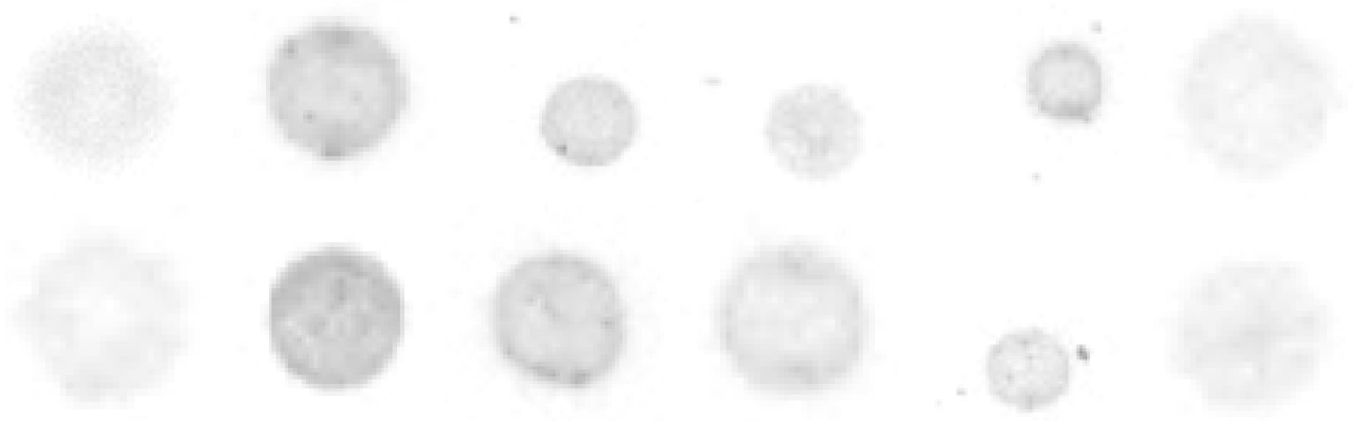

Figure 11. Label: Collodaria globule

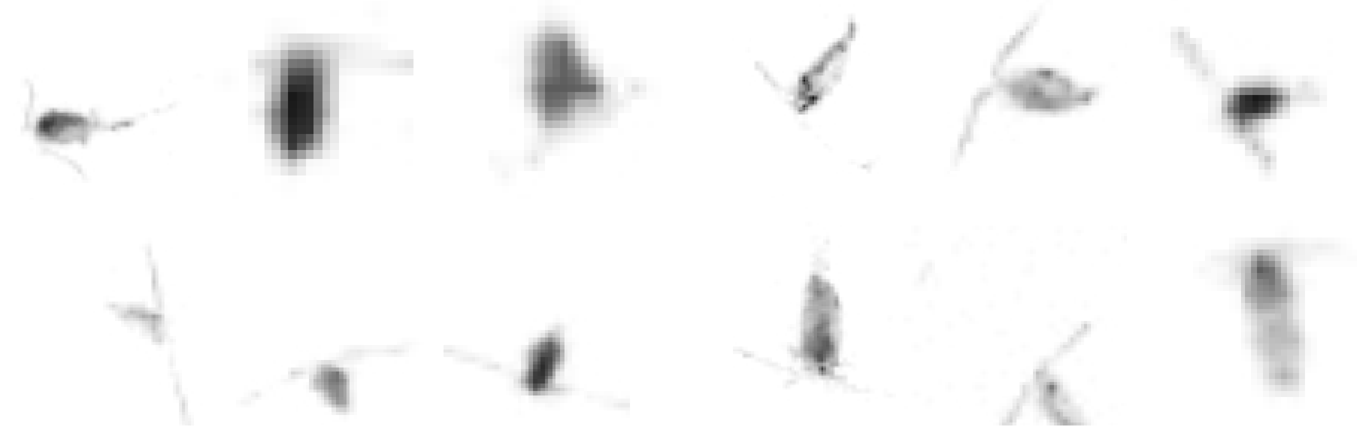

Figure 12. Label: Crust copepod

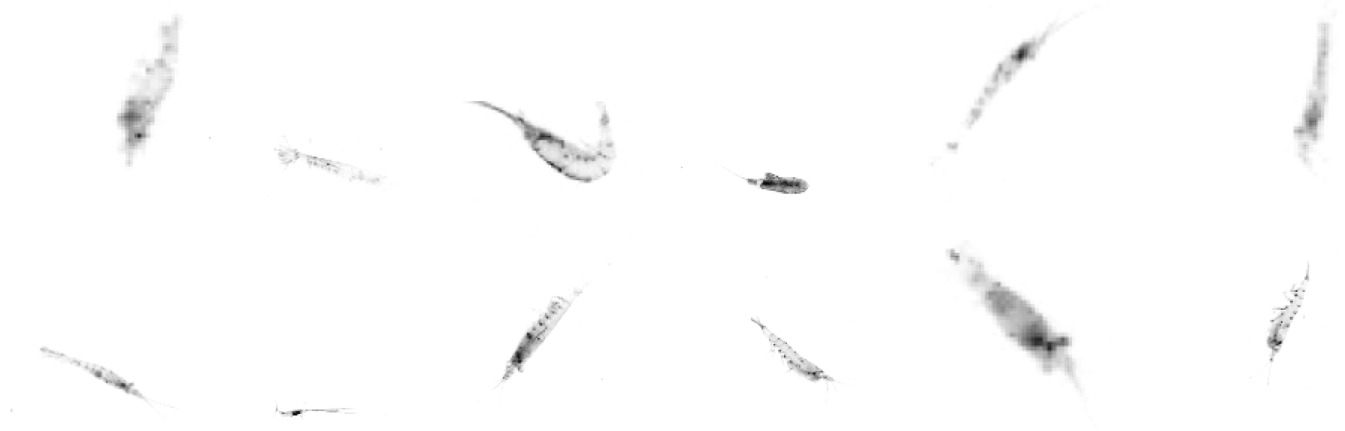

Figure 13. Label: Shrimp like

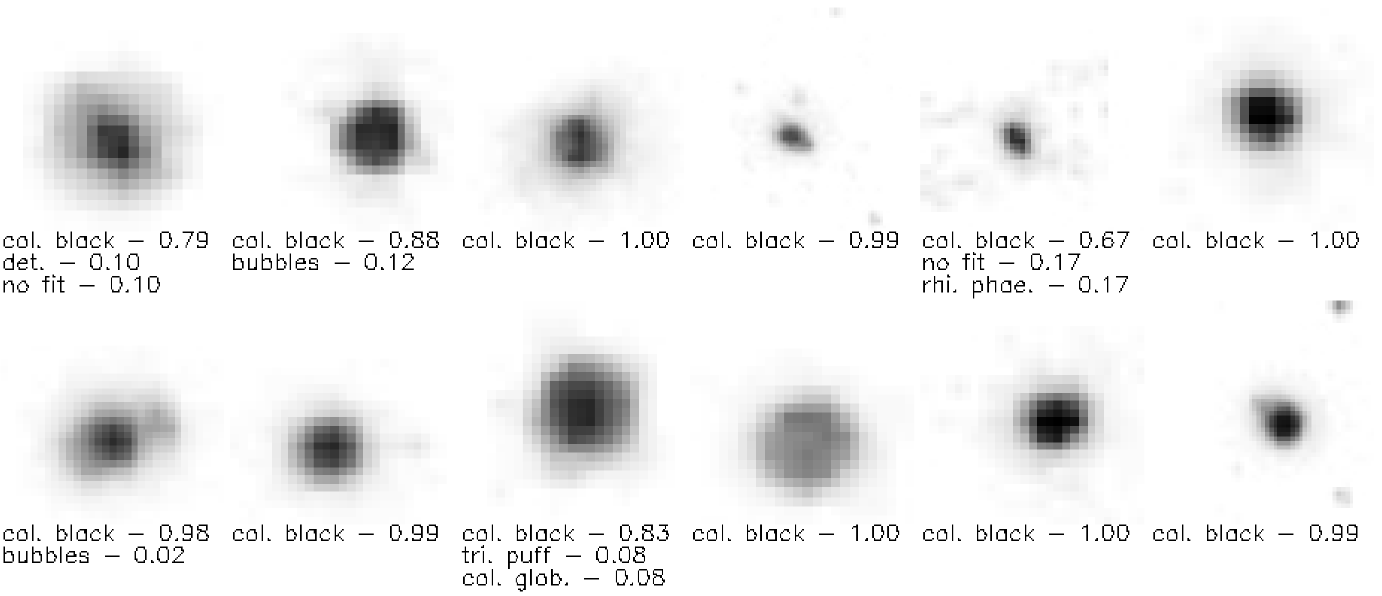

Figure 14. Cluster 01

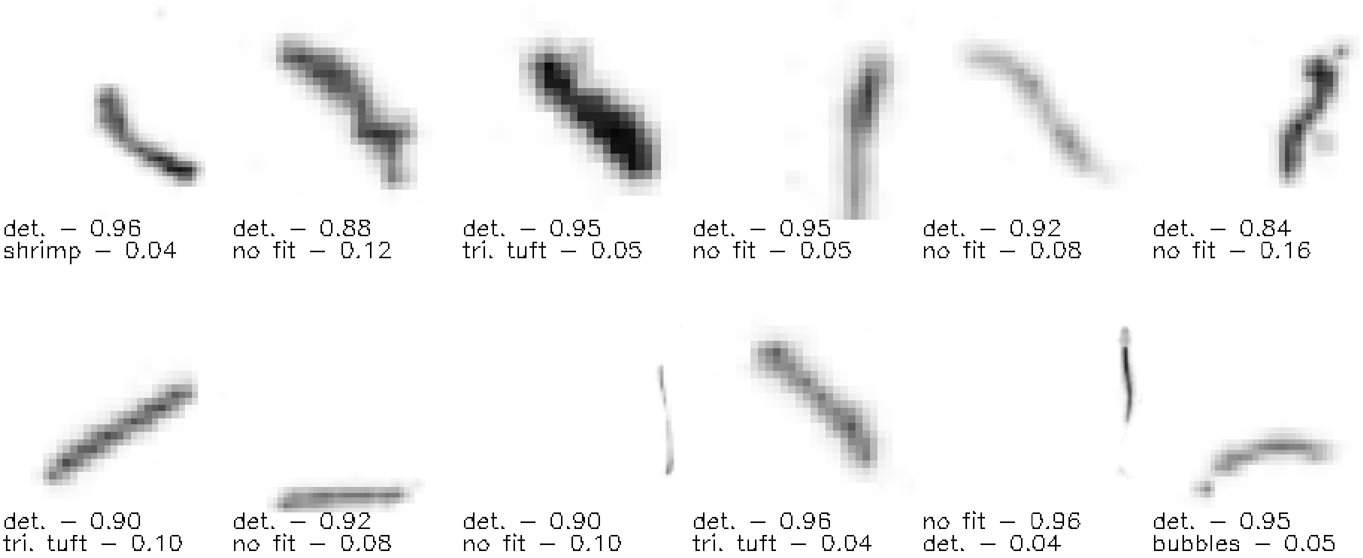

Figure 15. Cluster 02

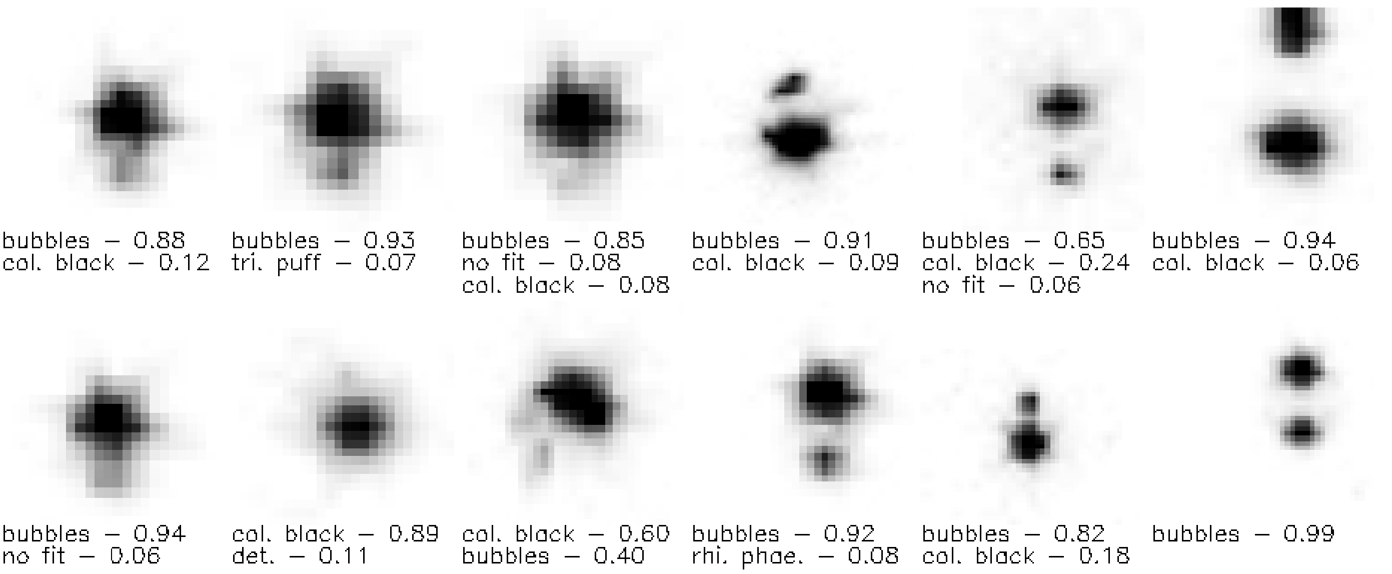

Figure 16. Cluster 07

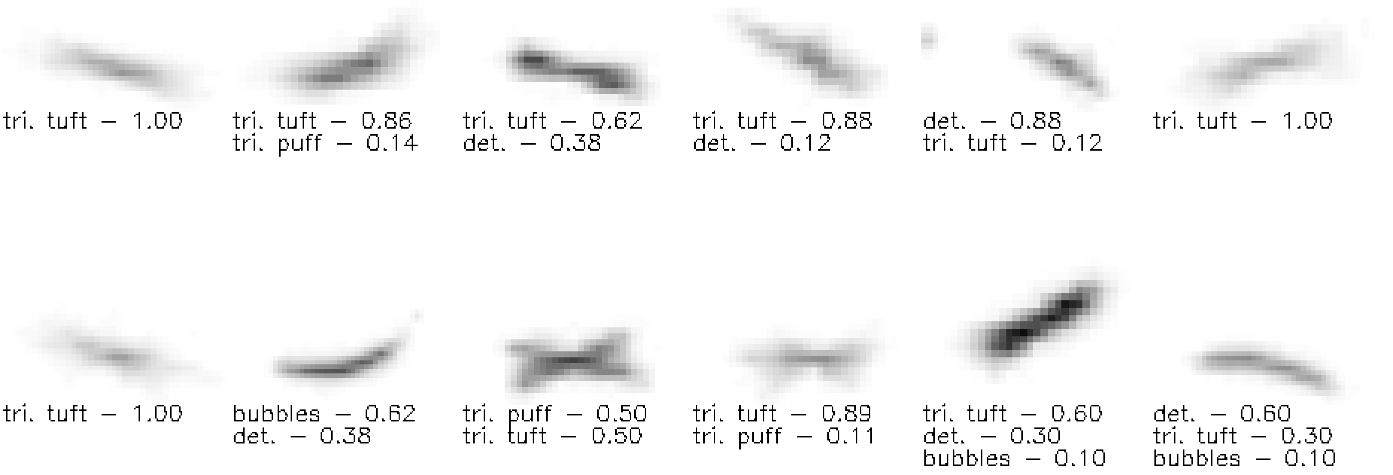

Figure 17. Cluster 09

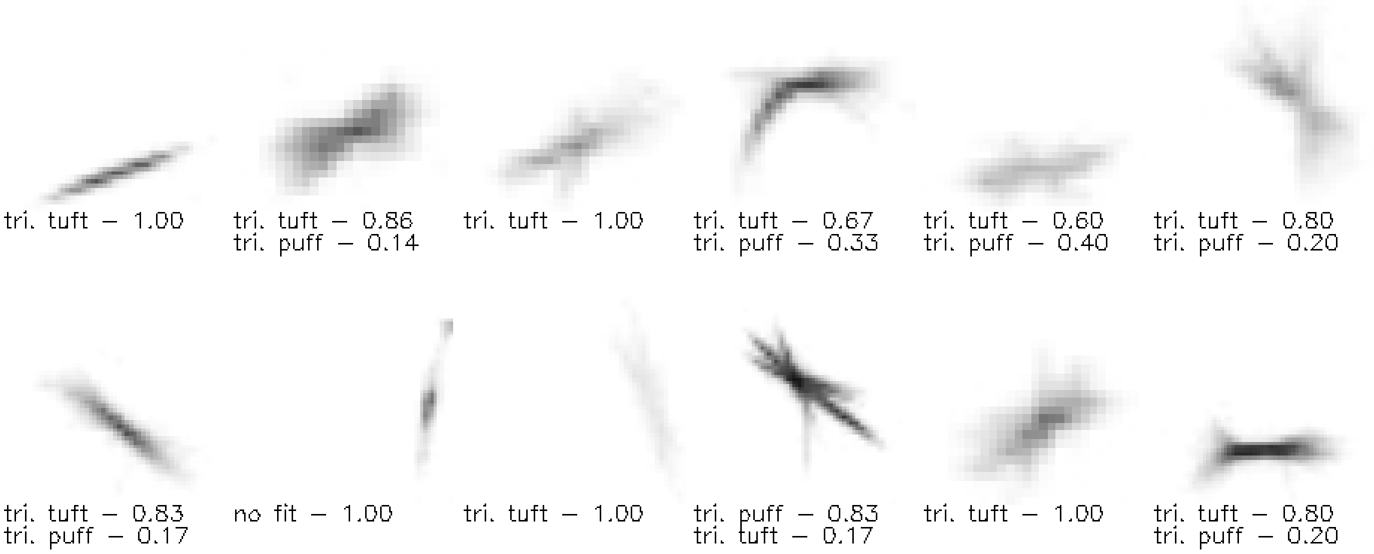

Figure 18. Cluster 10

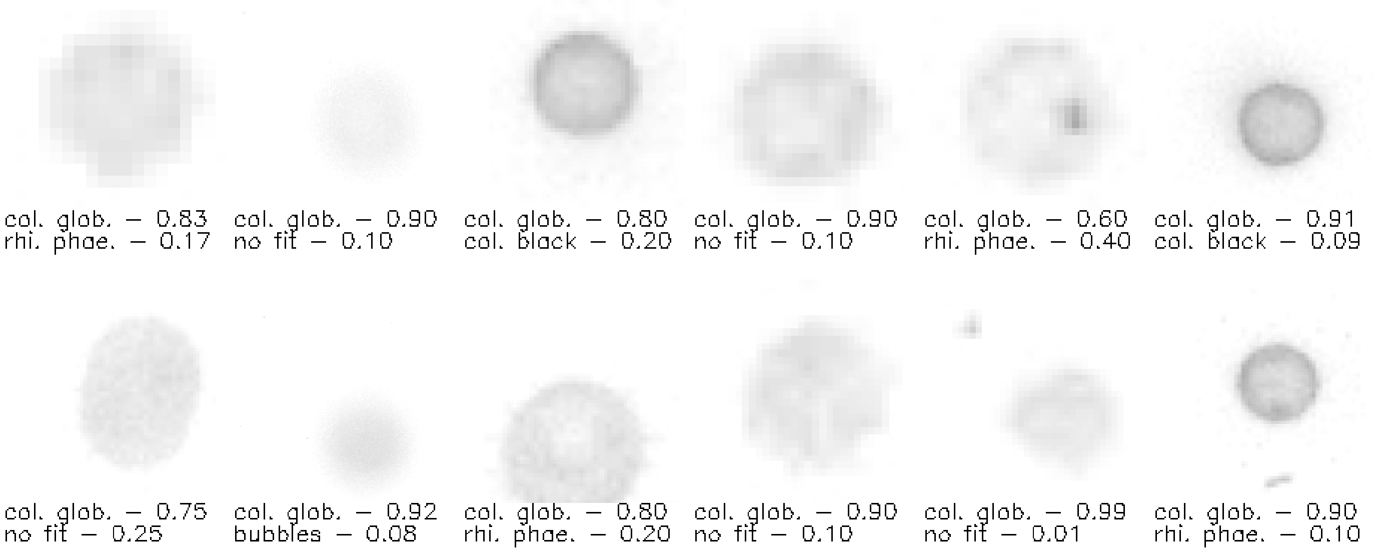

Figure 19. Cluster 11

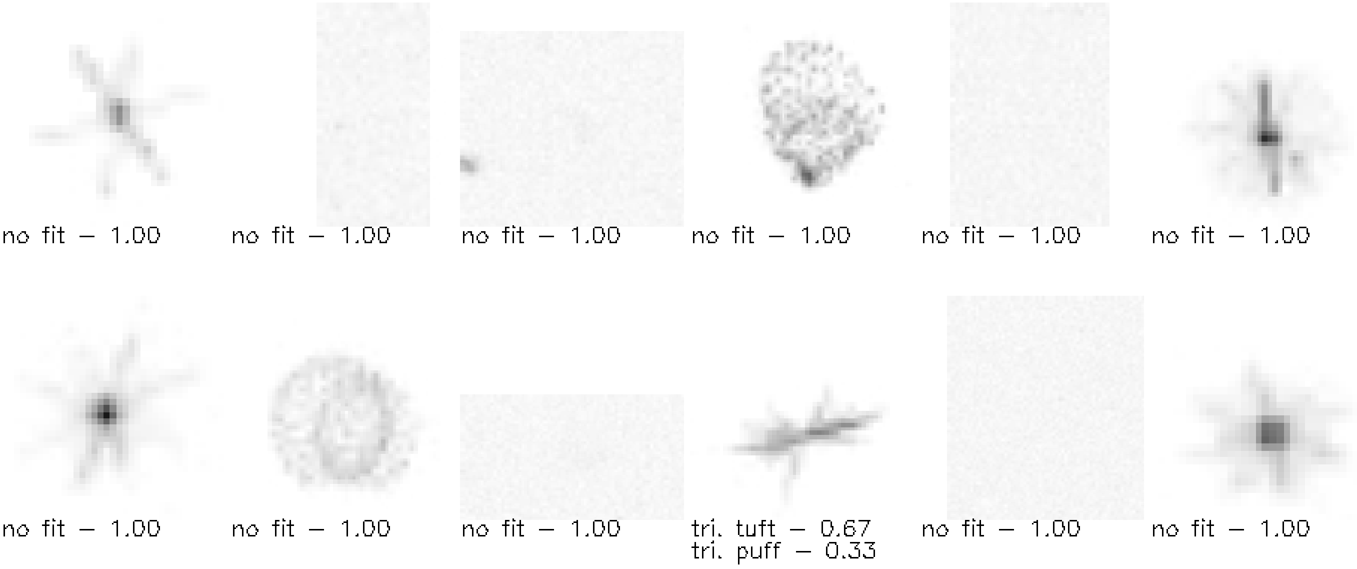

Figure 20. Cluster 12

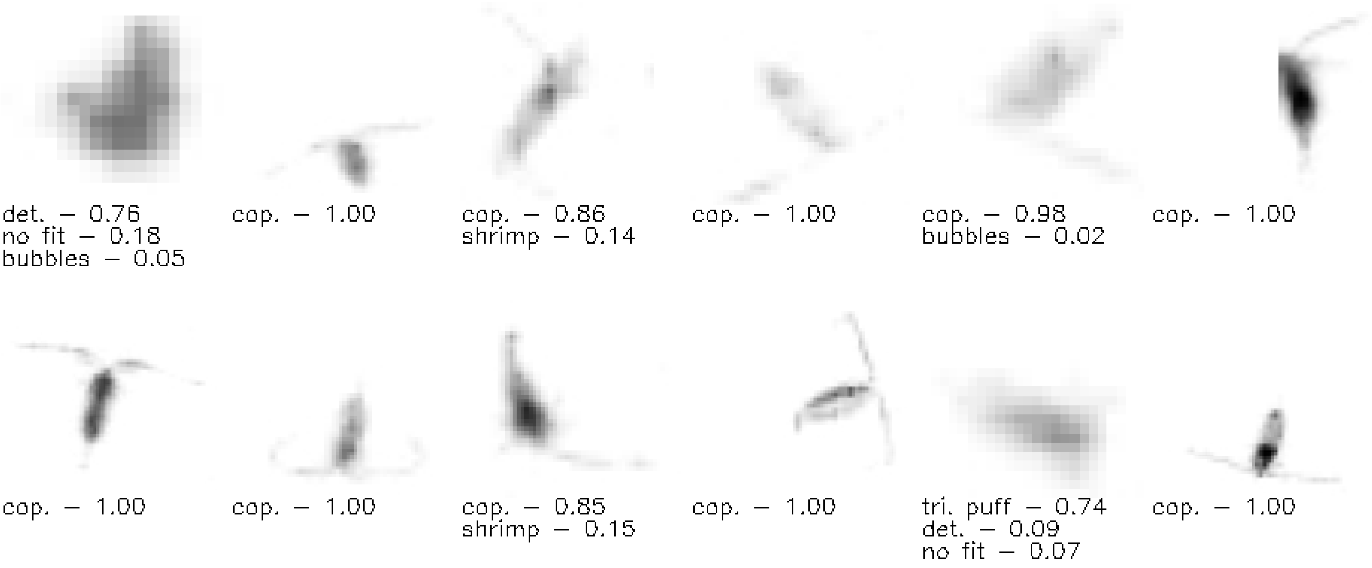

Figure 21. Cluster 24

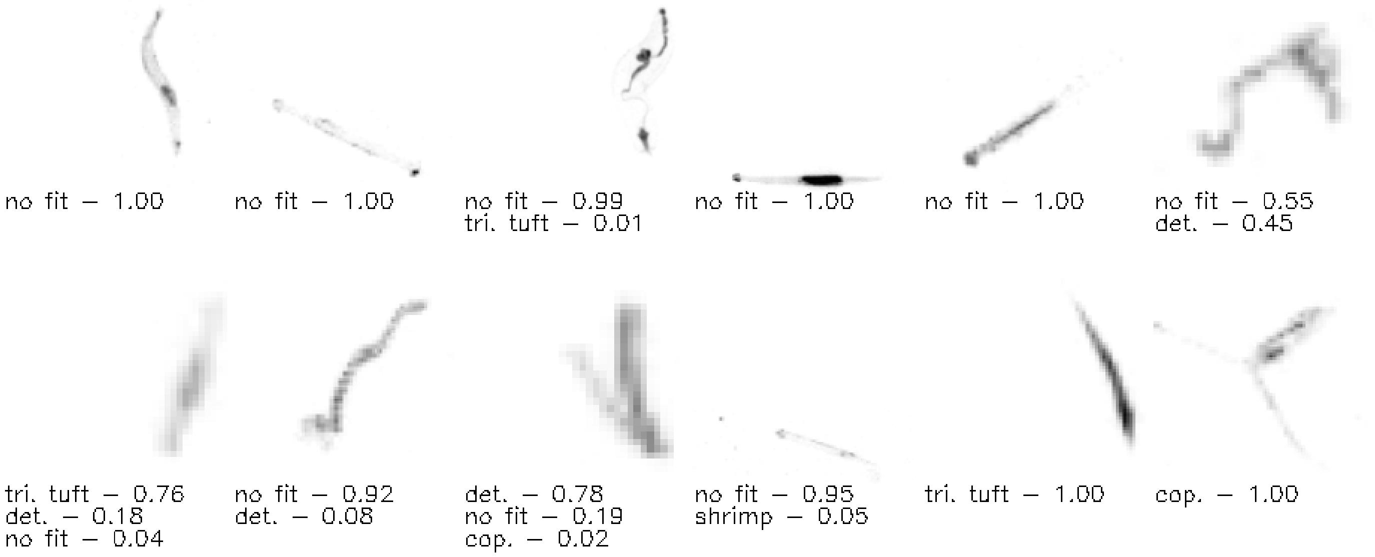

Figure 22. Cluster 26

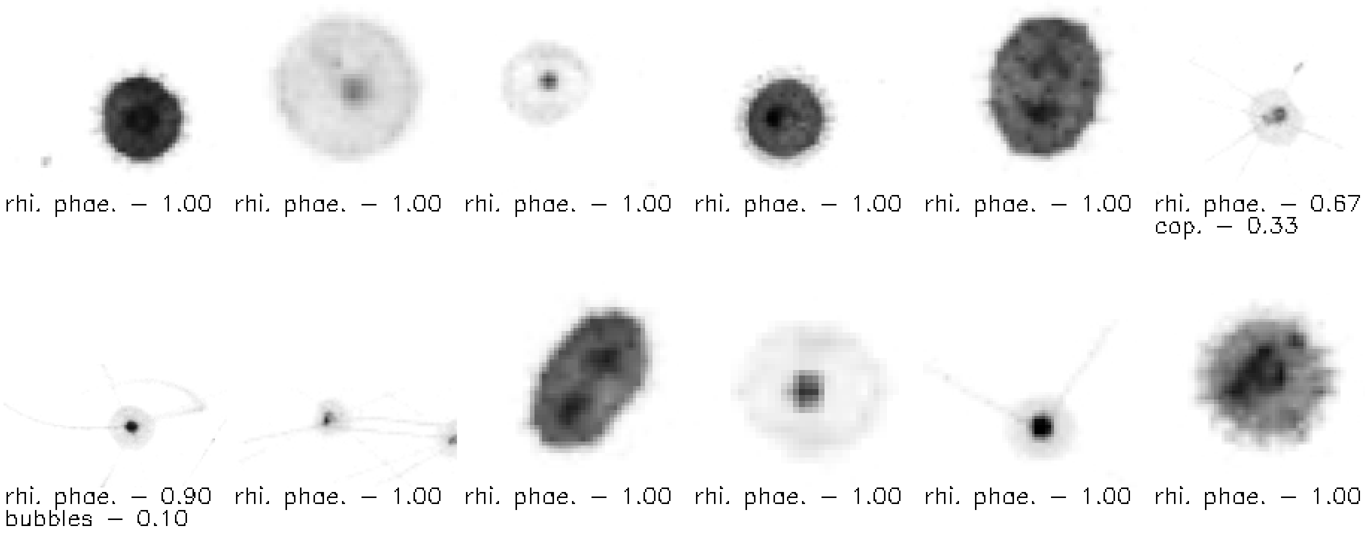

Figure 23. Cluster 31

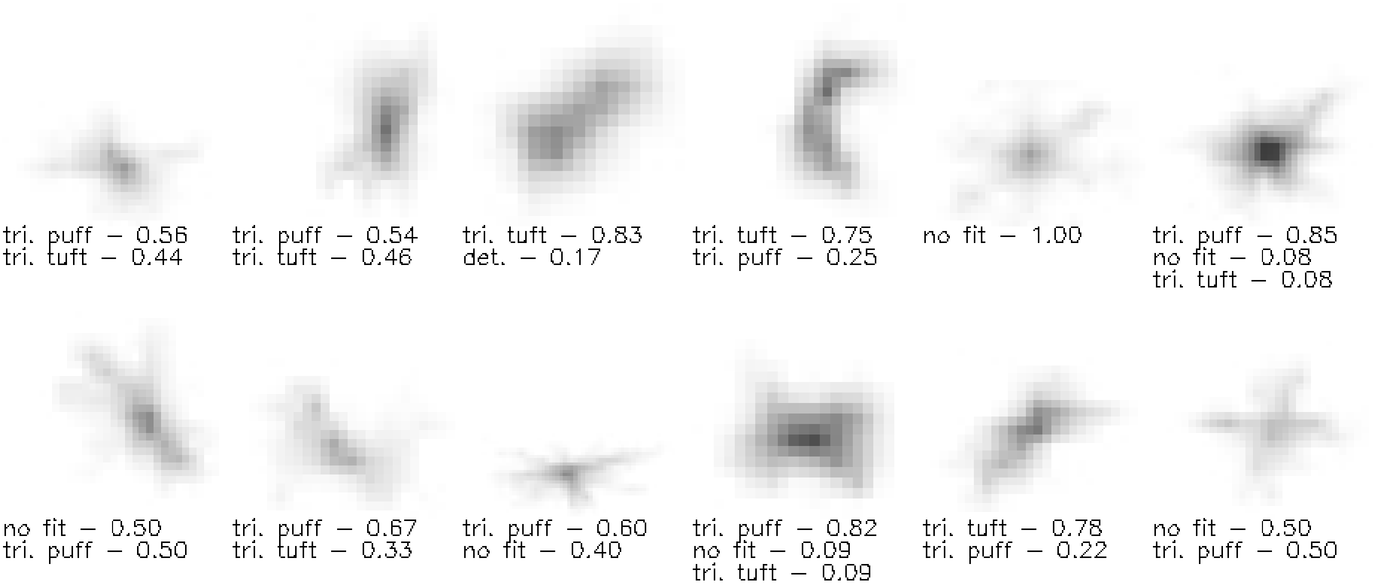

Figure 24. Cluster 36

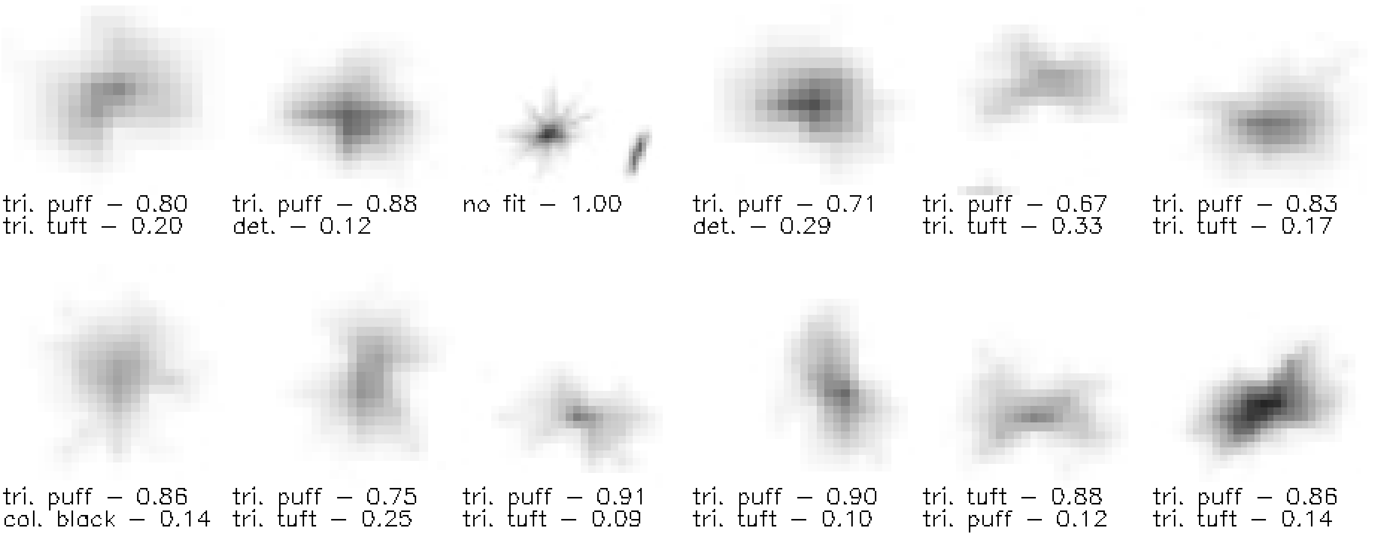

Figure 25. Cluster 41

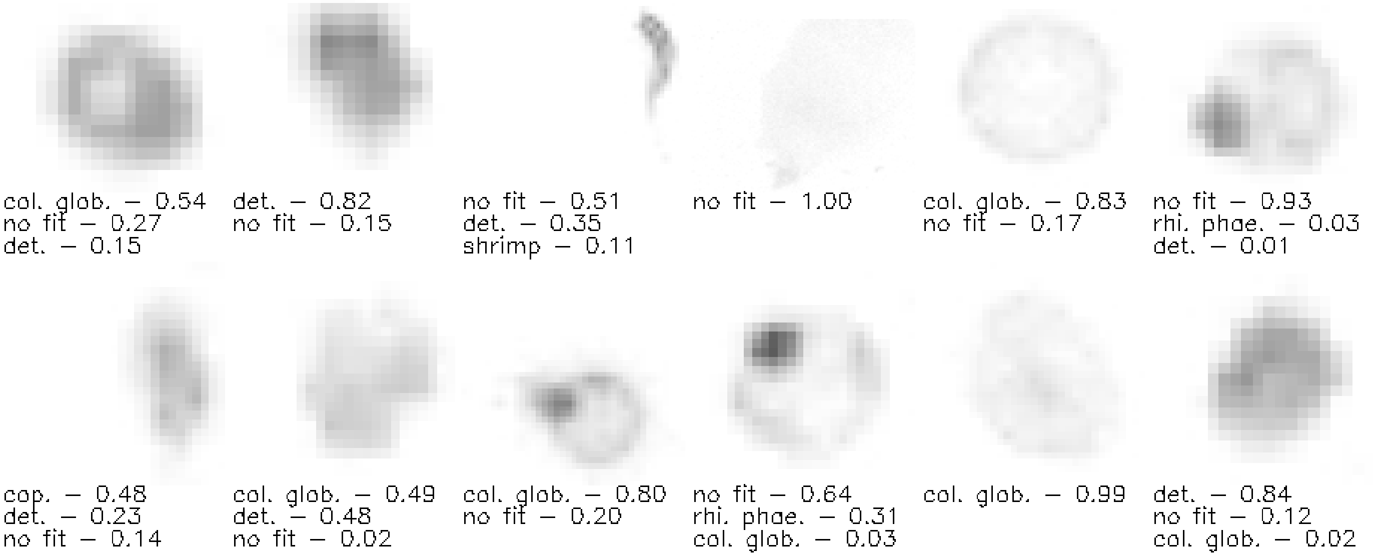

Figure 26. Cluster 43

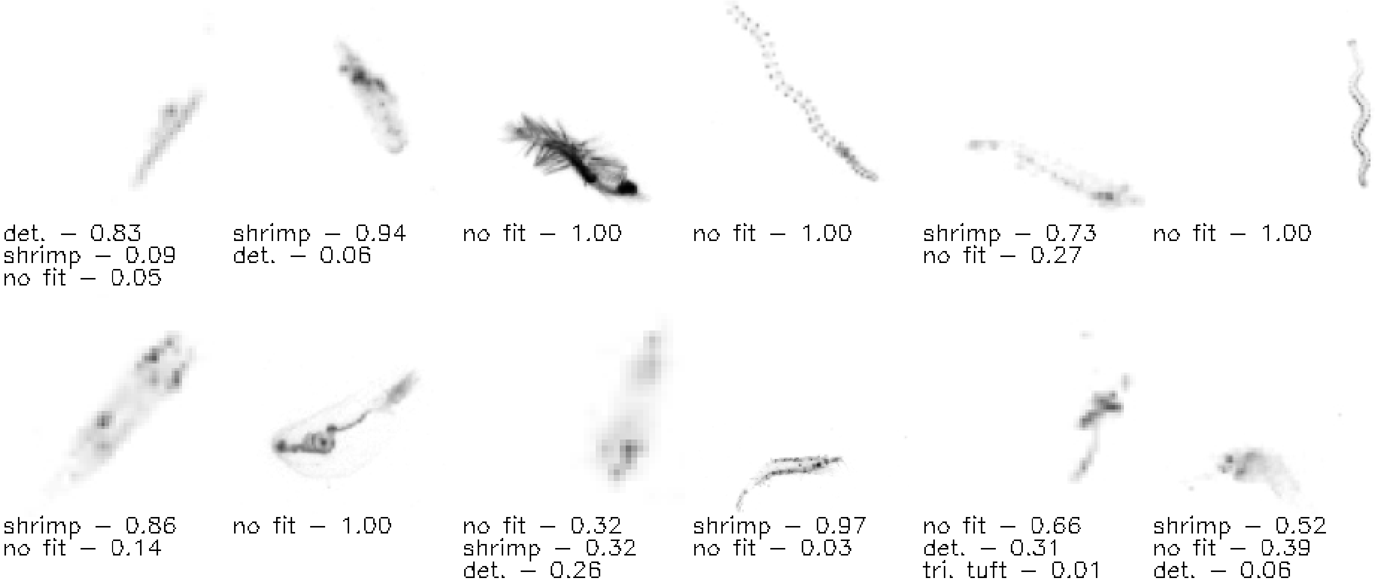

Figure 27. Cluster 44

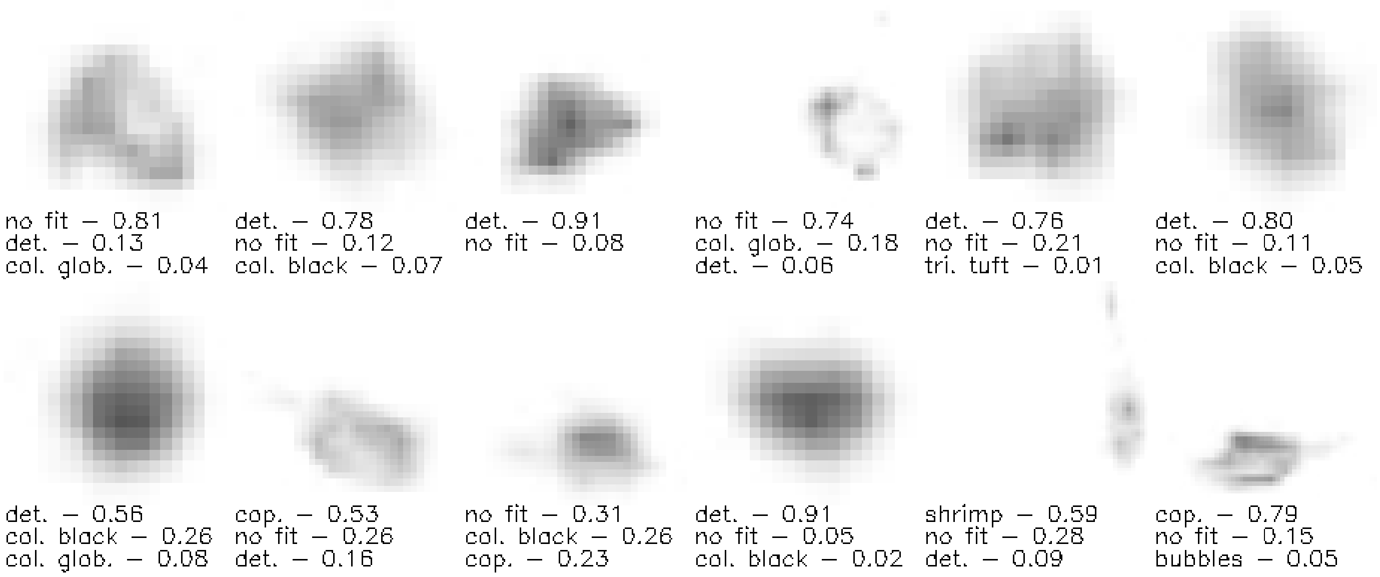

Figure 28. Cluster 45

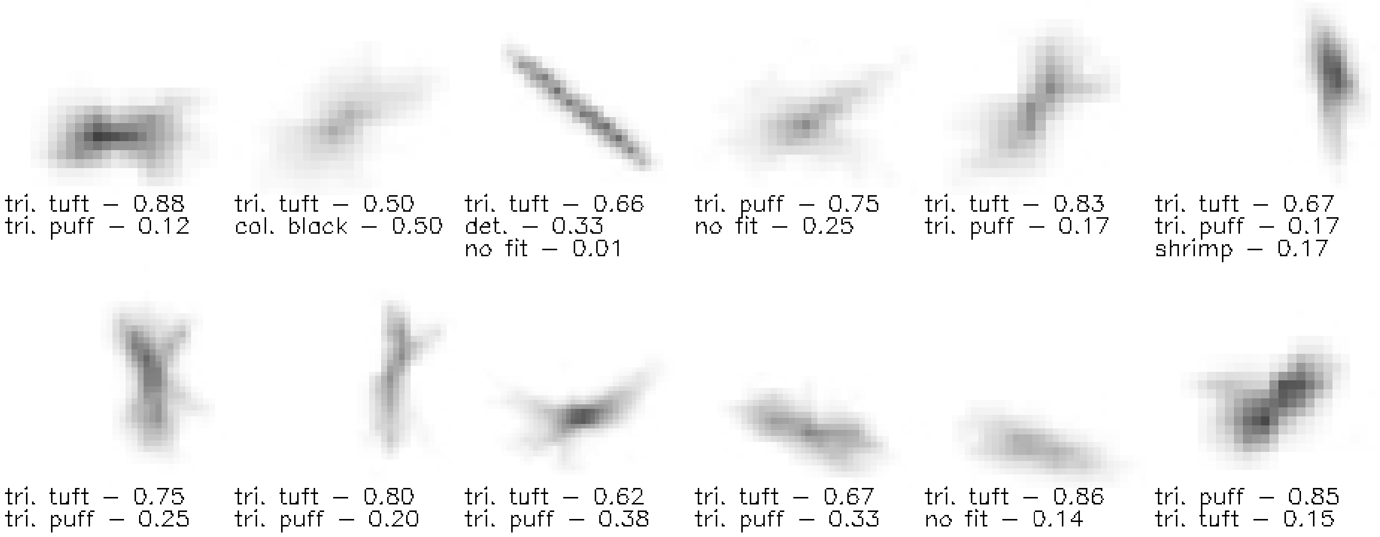

Figure 29. Cluster 47

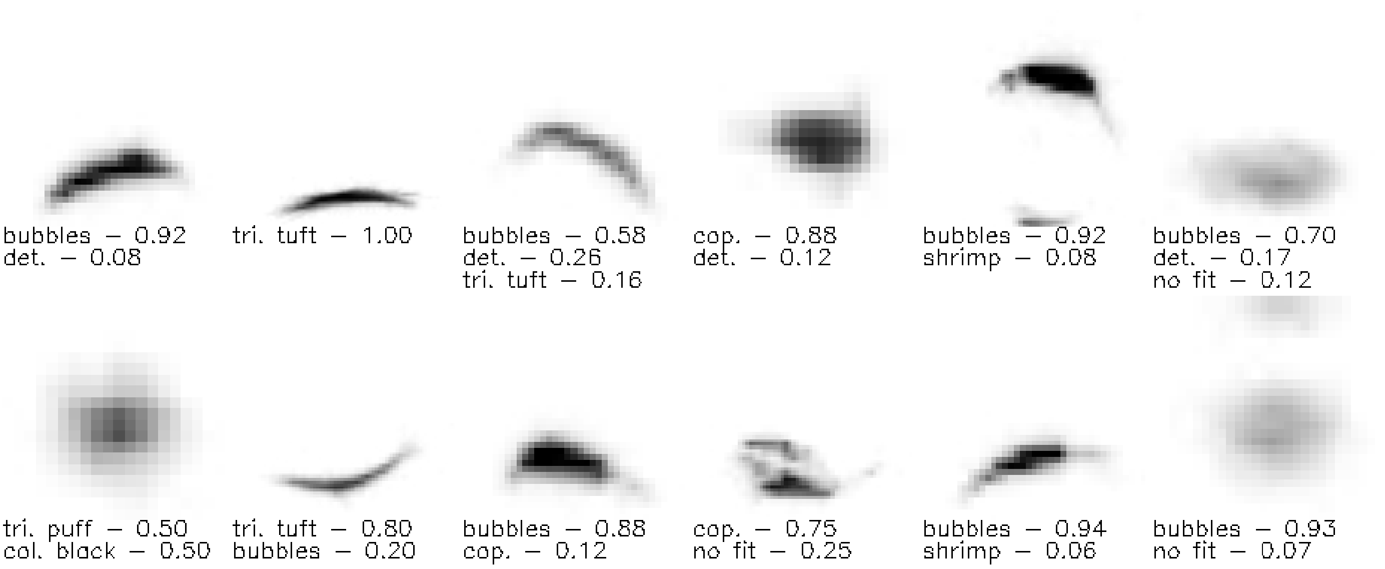

Figure 30. Cluster 51

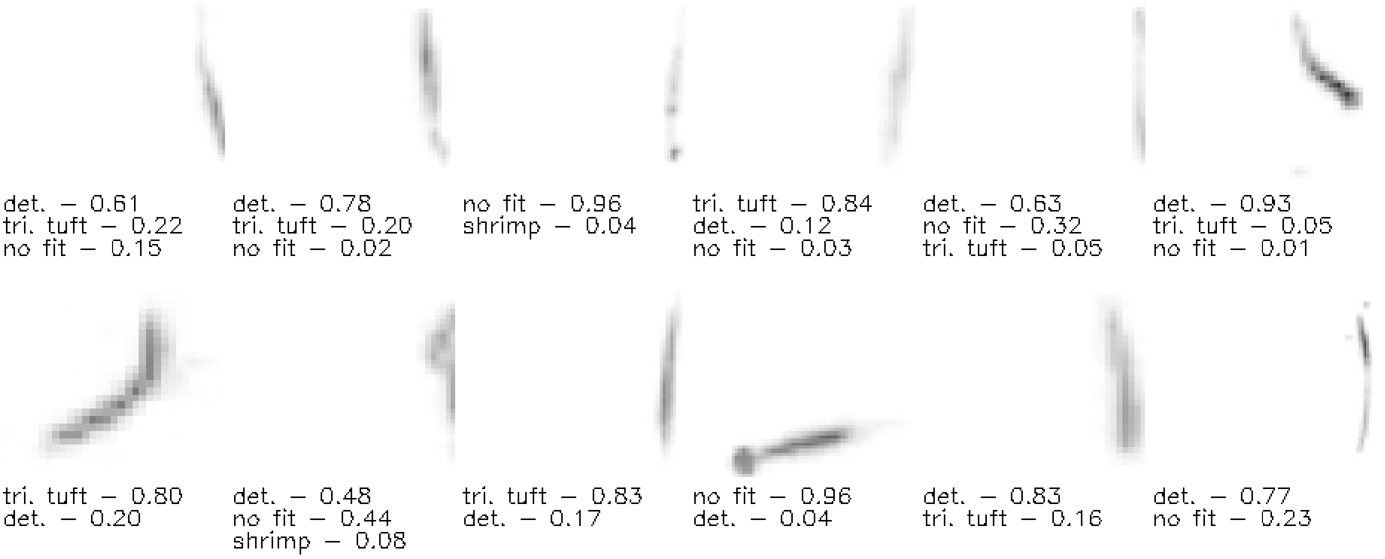

Figure 31. Cluster 52

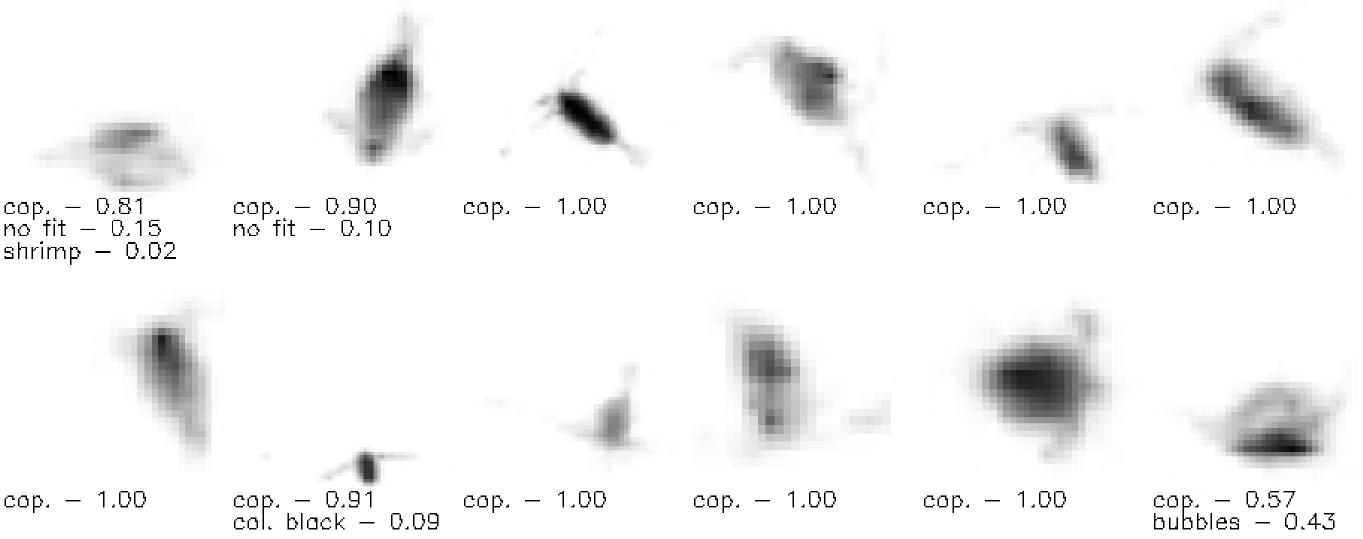

Figure 32. Cluster 53

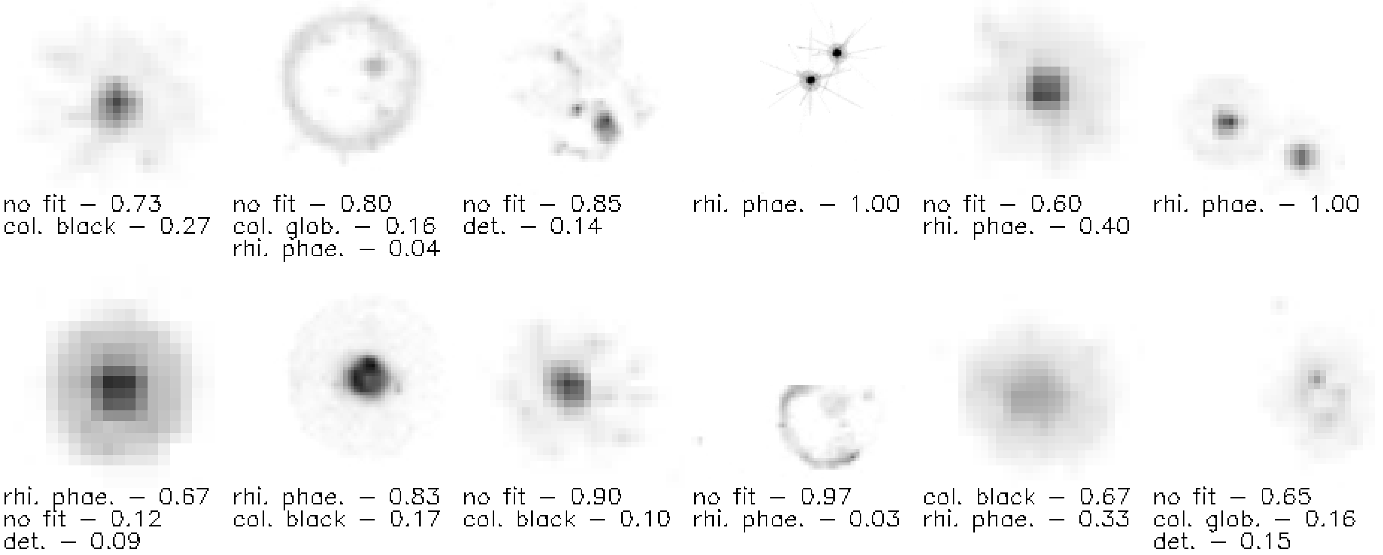

Figure 33. Cluster 59

1. Sohn, K.; Berthelot, D.; Li, C.L.; Zhang, Z.; Carlini, N.; Cubuk, E.D.; Kurakin, A.; Zhang, H.; Raffel, C. FixMatch: Simplifying Semi-Supervised Learning with Consistency and Confidence. *Advances in Neural Information Processing Systems 33 pre-proceedings (NeurIPS 2020)* **2020**.
2. Ji, X.; Henriques, J.F.; Vedaldi, A.; Ji, X.; Henriques, J.F.; Vedaldi, A. Invariant information clustering for unsupervised image classification and segmentation. Proceedings of the IEEE International Conference on Computer Vision, 2019, number Iic, pp. 9865–9874.
3. Chang, J.; Wang, L.; Meng, G.; Xiang, S.; Pan, C. Deep Adaptive Image Clustering. *2017 IEEE International Conference on Computer Vision (ICCV)* **2017**, pp. 5880–5888.
